# Elevated expression of mechanosensory polycystins in human carotid atherosclerotic plaques: association with p53 activation and disease severity

**DOI:** 10.1038/srep13461

**Published:** 2015-08-19

**Authors:** Aimilia Varela, Christina Piperi, Fragiska Sigala, George Agrogiannis, Constantinos H. Davos, Maria-Anastasia Andri, Christos Manopoulos, Sokrates Tsangaris, Efthimia K. Basdra, Athanasios G. Papavassiliou

**Affiliations:** 1 Department of Biological Chemistry, University of Athens Medical School, Athens 11527, Greece; 2 Cardiovascular Research Laboratory, Biomedical Research Foundation, Academy of Athens, Athens 11527, Greece; 3 Vascular Surgery Division, First Department of Propaedeutic Surgery, ‘Hippokrateion’ General Hospital, University of Athens Medical School, Athens 11527, Greece; 4 First Department of Pathology, ‘Laikon’ General Hospital, University of Athens Medical School, Athens 11527, Greece; 5 Laboratory of Biofluid Mechanics and Biomedical Engineering, School of Mechanical Engineering, National Technical University of Athens, Athens 15780, Greece

## Abstract

Atherosclerotic plaque formation is associated with irregular distribution of wall shear stress (WSS) that modulates endothelial function and integrity. Polycystins (PC)-1/-2 constitute a flow-sensing protein complex in endothelial cells, able to respond to WSS and induce cell-proliferation changes leading to atherosclerosis. An endothelial cell-culture system of measurable WSS was established to detect alterations in PCs expression under conditions of low- and high-oscillatory shear stress *in vitro*. PCs expression and p53 activation as a regulator of cell proliferation were further evaluated *in vivo* and in 69 advanced human carotid atherosclerotic plaques (AAPs). Increased PC-1/PC-2 expression was observed at 30–60 min of low shear stress (LSS) in endothelial cells. Elevated PC-1 expression at LSS was followed by p53 potentiation. PCs immunoreactivity localizes in areas with macrophage infiltration and neovascularization. PC-1 mRNA and protein levels were significantly higher than PC-2 in stable fibroatherotic (V) and unstable/complicated (VI) AAPs. Elevated PC-1 immunostaining was detected in AAPs from patients with diabetes mellitus, dyslipidemia, hypertension and carotid stenosis, at both arteries (50%) or in one artery (90%). PCs seem to participate in plaque formation and progression. Since PC-1 upregulation coincides with p38 and p53 activation, a potential interplay of these molecules in atherosclerosis induction is posed.

Wall shear stress (WSS) in vascular endothelium constitutes a primary regulator of endothelial function with both atheroprotective as well as atherosclerotic properties depending on its high or low range, respectively^1^,^2^. Among several mechanosensitive molecules that have been proposed to respond to WSS changes, the transmembrane proteins polycystin 1 (PC-1) and polycystin 2 (PC-2) are well-recognized^3^. PCs belong to the family of transient receptor potential channels (TRPP) that function as plasma membrane receptors in the membranes of intracellular organs. There is evidence that PC-1 requires PC-2 to form a mechanosensor complex that is important for cilia movement as well as for the development of kidney, skeletal muscle and heart.

PCs have been initially characterised as fluid shear stress sensors of kidney epithelial cells in Autosomal Dominant Polycystic Kidney Disease (ADPKD). Mutations in *PDK1* gene that encodes for PC-1 result in the fluid-filled cysts that characterize ADPKD and leads to early onset of renal failure as well as to severe cardiovascular complications^4^.

PCs have been found localized in several cell types including osteoblasts, cardiac myocytes and endothelial cells acting primarily as transmembrane mechanotransduction molecules that regulate cellular function, proliferation and apoptosis^5^,^6^,^7^,^8^,^9^,^10^,^11^,^12^,^13^,^14^,^15^.

Previous studies indicate that proper localisation and function of PC-1 is required for the mechanosensory function of primary cilia in endothelial cells^16^. In addition, cells with mutation of *PKD1* are unable to transmit extracellular shear stress into intracellular calcium signalling and nitric oxide synthesis suggesting a critical role of PC-1 in sensing and transducing fluid shear stress into biochemical response^17^,^18^,^19^,^20^,^21^,^22^,^23^,^24^,^25^. We have previously shown PCs activation at the early shear stress signalling events in the vascular endothelium of an *in vivo* model of partial carotid artery stenosis^26^.

There is evidence that a downstream target of PC-1 signalling is the tumour suppressor protein p53, a critical regulator of cell proliferation and apoptosis. PC-1 has been found to regulate a G1 checkpoint in HEK293 cells via p53 activation^27^. Moreover, mouse PKD-1 knockout cells can undergo immortalized cell proliferation that is associated with downregulation of PC-1–JNK–p53 signalling^10^. It has been suggested that PC-1 and p53 participate in an autofeedback pathway which functions to tightly regulate their expression, and aberrant expression of either protein leads to impaired development^28^,^29^. Furthermore, p53 as a determinant of cell fate has been implicated in the development of atherosclerosis^30^ and vascular smooth muscle cell (VSMC) apoptosis^31^. Biomechanical stress has been found to induce p53 activation in SMCs, leading to apoptosis via p38 mitogen-activated protein kinase (MAPK) signalling^31^,^32^.

Taken together this data, we hypothesized the implication of PCs-induced mechanotransduction mechanisms in the development of atherosclerosis. The present study investigates variations in endothelial PCs expression under conditions of low and high oscillatory shear stress and links to p53 activation *in vitro* and *in vivo*. In addition, a detailed analysis of PCs expression levels is performed in stable and unstable human carotid atherosclerotic plaques (AAPs) in relation to p53 activation, plaque histopathology and degree of carotid stenosis.

## Results

### Low shear stress (LSS) upregulates PC-1 and PC-2 protein expression and activates p53

**Figure 1:**
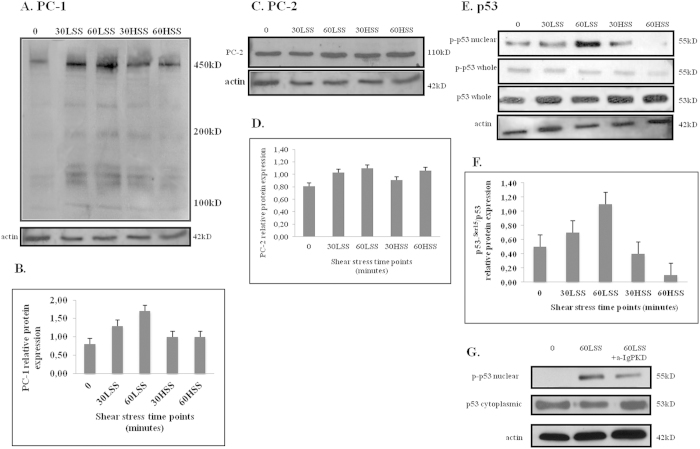
Western immunoblots showing protein expression levels of PC-1 (**A**), PC-2 (**C**) and p53 (**E**) under 30 and 60 min of low shear stress (LSS) and high shear stress (HSS) in an *in vitro* model of cultured endothelial cells compared to static controls (0). The densitometric quantification of protein levels (normalized to actin levels) is shown (**B**,**D**,**F**). (**G**) Western immunoblots showing the effect of endothelial cells pretreatment with anti-IgPKD inhibitory antibody on the protein expression levels of phospho(p)-p53 and p53 at 60 min of LSS compared to static control. All experiments were performed at least three times and representative results as well as corresponding quantification data of one experiment are shown.

Using an *in vitro* cell culture model, we investigated the acute effects of oscillatory shear stress on PC-1 and -2 expression. HUVEC cultures were equilibrated in fluid shear stress of 0, 4 and 10 dynes/cm^2^ for 30 and 60 min. Interestingly, PC-1 expression (~450 kDa) was found elevated at 30 and 60 min of low oscillatory shear stress compared to high oscillatory shear stress (Fig. 1A,B). Two secondary truncated species of PC-1 (200 kD, 100 kD) were observed in cells exposed to shear stress compared to a single full-length PC-1 species (450 kD) observed in static control cells, indicating the proteolytic cleavage of PC-1 under fluid shear stress.

PC-2 expression was increased at LSS with higher value at 60 min of LSS exposure. A similar expression pattern was also observed under high shear stress (HSS) conditions (Fig. 1C,D).

Additionally, the transcription factor p53 (phosphorylated hence activated form; phospho(p)-p53) migrated to the nucleus at 30 and 60 min of low oscillatory shear stress with higher activation observed at 60 min of LSS (Fig. 1E,F). This effect was significantly reduced (3.5 fold i.e. 70% reduction) when the cells were incubated with an inhibitory antibody against the PC-1 extracellular domain (anti-IgPKD Ab) three hours prior to exposure to low LSS (Fig. 1G), indicating a direct functional interaction of these two molecules in LSS signalling on vascular endothelium.

### Upregulation of PC-1 and PC-2 protein expression and p53 activation in LSS regions of vascular endothelium *in vivo*

**Figure 2:**
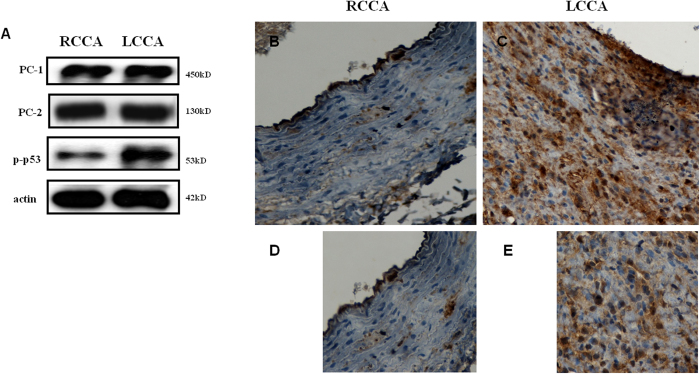
Western immunoblots showing protein expression levels of PC-1, PC-2, phospho(p)-p53 (**A**) in right common carotid artery (RCCA) and left common carotid artery (LCCA) of the partial carotid stenosis experimental model. Immunohistochemical analysis of p-p53 in RCCA (**B**,**D**) and LCCA (**C**,**E**) tissues. Higher immunoreactivity for p-p53 is observed in the endothelial cells at the low shear stress (LSS) region of LCCA.

Using a partial carotid artery stenosis experimental animal model previously established in our laboratory suitable for studying LSS signalling on vascular endothelium^26^, we proceeded to investigate potential associations between polycystins expression and p53 activation *in vivo*. Increased PC-1 and PC-2 protein expression was observed in the cytoplasm of endothelial cells of LSS region generated after partial ligation of the left common carotid artery (LCCA) compared to physiological shear stress area at the right common carotid artery (RCCA). Furthermore, western immunoblotting analysis revealed elevated p-p53 levels by three fold at the LCCA compared to the RCCA (Fig. 2A). Similarly, increased p-p53 immunoreactivity was observed in endothelial cells of LCCA tissue compared to RCCA (Fig. 2B–E).

### Elevated mRNA PKD1 levels in advanced atherosclerotic plaques

**Figure 3:**
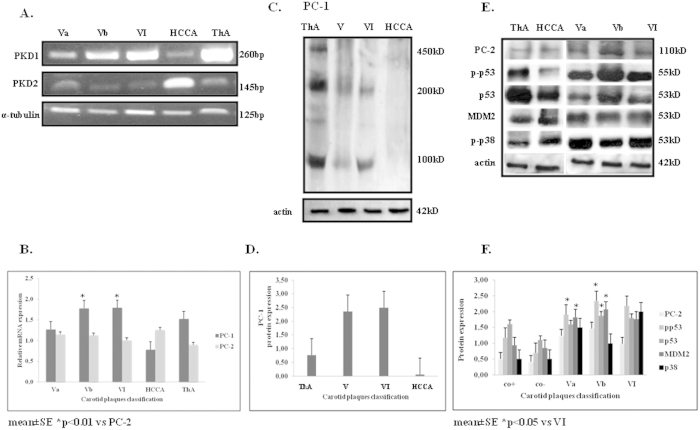
*PKD1* and *PKD2* mRNA levels (**A**) in stable and unstable atherosclerotic plaques as evaluated by RT-PCR analysis. Total RNA tissue extracts from human thyroid arteries (ThAs) and human common carotid arteries (HCCAs) were used as positive and negative controls, respectively. The densitometric quantification of mRNA levels (normalized to *α-tubulin*) is shown (**B**). Western immunoblots showing protein expression levels of PC-1 (**C**), PC-2, phospho(p)- p53/p53/MDM2, phosphor(p)-p38 (**E**) in stable and unstable atherosclerotic plaques. Tissue protein extracts from ThAs and HCCAs were used as positive and negative controls, respectively. The densitometric quantification of protein levels (normalized to actin levels) is shown (**D**,**F**).

In order to investigate the presence of PC-1 and PC-2 in advanced atherosclerotic plaques, we first determined *PKD1* and *PKD2* mRNA levels in 22 human tissues. An upregulation of *PKD1* mRNA was observed both in stable fibroatherotic plaques (Va, Vb) and unstable atherosclerotic plaques (VI) (Fig. 3A,B). Significantly lower *PKD2* mRNA levels (*P* = 0.01) were detected in both atherosclerotic plaque types (Fig. 3A,B).

### Increased PC-1 and PC-2 expression levels in advanced atherosclerotic plaques

Detection of protein expression levels of PC-1 and PC-2 in atherosclerotic plaques revealed increased PC-1 protein levels (~100 kDa) in stable and unstable atherosclerotic plaques compared to controls (Fig. 3C,D). Interestingly, we observed only the secondary truncated species of 200 and 100 kDa in these samples, suggesting the proteolytic cleavage of PC-1 during the atherosclerotic process. PC-2 expression levels were also found elevated in stable and unstable atherosclerotic plaques compared to controls (Fig. 3E,F).

### Increased activation of p38 and p53 in advanced atherosclerotic plaques

Activation of p53 was also evaluated in the same atherosclerotic plaques as a potential downstream target of PC-1 upregulation. Indeed, p-p53 levels were higher in stable and unstable atherosclerotic plaques compared to controls (Fig. 3E,F). In addition, the p-p53:p53 ratio was 2.1 compared to 1.2 for thyroid artery and 1.3 for HCCAs. In accordance, activation of p38, a potential mediator of p53 phosphorylation was also found increased in stable and unstable atherosclerotic plaques (Fig. 3E,F).

Furthermore, the levels of mouse double minute 2 homolog (MDM2), the principal cellular antagonist of p53, were found decreased compared to p-p53 levels in atherosclerotic plaques (Fig. 3E,F).

### Immunohistochemical localization of PC-1 and PC-2 expression in advanced atherosclerotic plaques

The distribution of PC-1 and PC-2 expression was further evaluated immunohistochemically in type V and VI carotid plaques of 69 patients. PCs immunoreactivity was distributed focally, with a preferential localization in defined components of the plaque.

**Figure 4:**
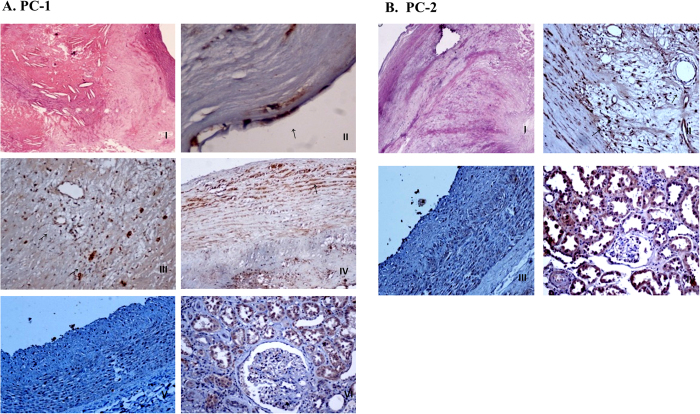
Immunohistochemical detection of PC-1 (**A**) and PC-2 (**B**) in atherosclerotic plaques. Positive staining is observed in the cytoplasm of ECs (**A.II**), in the cytoplasm of SMCs (**A.IV**,**B.II**), in the cytoplasm of macrophages/foam cells (**A.III**,**B.II**) and in the ECs of neovascularized areas (**A.III**,**B.II**). Tissue specimens from human kidney (**A.VI**,**B.IV**) and coronary artery (**A.V**,**B.III**) were used as positive and negative controls, respectively. [**A.II**: magnification × 400; **A.III-VI** and **B.II-IV**: magnification × 200].

Only PC-1 expression was specifically observed in the cytoplasm of endothelial cells of the intima (Fig. 4A.II) whereas both PC-1 and PC-2 immunostaining was observed in the cytoplasm of SMCs (Fig. 4A.IV,B.II), macrophages (Fig. 4A.III,B.II) and endothelial cells of neovascularized areas (Fig. 4A.III,B.II).

**Figure 5:**
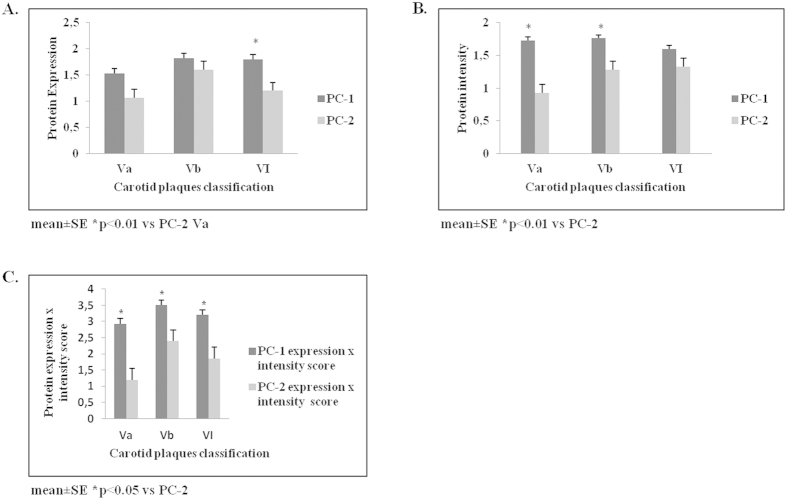
Quantification diagrams showing variations in protein expression (**A**), staining intensity (**B**) or protein expression multiplied by intensity score (**C**) of PC-1 and PC-2 are depicted (see also Fig. 4).

PC-1 and PC-2 protein expression (as well as expression × intensity score) was elevated in stable (Vb) and unstable/complicated (VI) atheromatic plaques. However, PC-1 expression was higher than PC-2 in both types (*P* = 0.01; Table 1, Fig. 5A–C).

Further analysis of PC-1 staining intensity revealed higher PC-1 levels compared to PC-2, in patients with carotid stenosis up to 50% at both arteries due to atherosclerotic plaque (*P* = 0.01) as well as in patients with carotid stenosis up to 90% in one of the two arteries (*P* = 0.008). Additionally, increased PC-1 intensity was observed at the plaques of patients with diabetes (*P* = 0.05), hypertension (*P* = 0.003) and dislipidemia (*P* = 0.003, Table 2).

## Discussion

The present study investigates for the first time the implication of the mechanosensitive molecules PC-1 and PC-2 in the induction of atherosclerosis and plaque formation. Hemodynamic forces and particularly low WSS in blood vessels have been associated with atherosclerotic plaque formation through modulation of endothelial gene expression and function^1^,^2^,^3^,^33^,^34^,^35^,^36^.

Using an *in vitro* endothelial cell culture system of measurable WSS to investigate the direct effects of specific range oscillating shear stress in PCs expression, we have detected elevated expression of PC-1 at LSS conditions compared to HSS. Although a high amount of functional full-length PC-1 was maintained, fluid shear stress in both cases was found to induce proteolytic cleavage of PC-1 and this cleavage has been previously associated with the mechanical fluid stimulus^16^,^37^. Previous studies exposing endothelial cells to LSS (1-7 dynes/cm^2^) have shown a similar pattern for PC-1 expression which however did not alter cell responsiveness and function. However, higher shear stress (over 7 dynes/cm^2^) was shown to disassemble cilia in endothelial cells and render them mechanoinsensitive^38^.

PC-2 expression was also increased at LSS conditions compared to static controls however, to a lesser extent than PC-1. Furthermore, a smaller reduction of PC-2 levels was observed at HSS, indicating the primary mechanosensory role of PC-1 under shear stress fluctuations. This is in accordance with our previous work showing that early shear stress signalling in vascular endothelium involves the activation of PC-1 and PC-2 in a partial carotid ligation animal model^26^. Activation of p53 under the same conditions of LSS that induce upregulation of PC-1 *in vitro* indicates a potential link of these two molecules in the regulation of cell proliferation. Furthermore, blocking of PC-1 extracellular domain with an anti-IgPKD antibody prior to exposure of endothelial cells to 60 min of LSS reduced activated p53 (p-p53) levels by 3.5 fold (70%), indicating a specific functional interaction of these two molecules under LSS conditions. This interaction was further reproduced in the *in vivo* experimental model of carotid stenosis, where both PC-1/PC-2 and p-p53 were found upregulated at the endothelial cells of the LSS region.

The above data were further expanded with evaluation of human atherosclerotic plaques of differential stability. Semiquantitative analysis of atherosclerotic tissue showed a significant increase of PC-1 mRNA and protein levels compared to PC-2 in stable Vb and unstable VI atherosclerotic plaques compared to fibroatheromatic ones (Va) suggesting that PC-1 may be implicated in the progression of atherosclerosis and plaque severity.

Immunohistochemical analysis of the atheromatous plaques revealed localization of PC-1 in the endothelial cells of the intima. Plaque formation starts with deposition of small cholesterol crystals in the intima and its underlying smooth muscle leading to proliferation of fibrous tissues, therefore, it is possible that PC-1 is implicated at the early disease stages. Furthermore, both PC-1 and PC-2 were found localized predominantly to areas with macrophage infiltration and neovascularization in the vicinity of atheromatous gruel. PC staining was evident in the cytoplasm of SMCs, macrophages and endothelial cells of the neovascularized areas. The association of PCs immunoreactivity with plaque components indicative of chronic inflammatory responses suggests a role for local inflammatory processes in the production of PCs within atherosclerotic lesions. A role of PCs in inflammation has been proposed by studies in genetically engineered animal models carrying mutations in *PKD* genes where prominent interstitial changes in the cystic kidney are largely attributed to inflammatory cell infiltration^39^,^40^. This is in accordance with human APKD, where the kidney disease phenotype is also characterized by interstitial changes including inflammation, apoptosis, proliferation, and fibrosis.

Recruitment of macrophages and T lymphocytes in the arterial wall, proliferation of smooth muscle cells, matrix formation and neovascularization are main characteristics of the chronic inflammatory events leading to atherosclerosis^41^. Detection of PCs immunoreactivity in VSMCs is in accordance with previous studies^21^,^22^,^23^,^42^ showing that exposure of endothelial cells to disturbed flow may also affect the expression of PCs in VSMCs. PKD2 has been shown to inhibit the activity of stretch-activated ion channels in VSMCs and increased PKD2 expression is associated with impaired arterial myogenic tone by altering the PKD1:PKD2 ratio^20^,^23^.

Macrophages being the principal inflammatory cells in atherosclerotic plaques, they are also known mediators involved in the transition of stable atherosclerotic lesions into active ones^43^,^44^. Therefore the presence of PCs immunostaining in macrophage-rich areas of atheromatous plaques, indicative of active inflammatory responses, supports the participation of PCs in inflammatory processes and their involvement in active coronary atherosclerotic lesions. Furthermore, the presence of PC immunoreactivity in endothelial cells of neovascularized areas, another histologic feature of chronic inflammatory response, implicate PCs to formation of plaque microvessels that contribute to plaque evolution or complication.

Carotid stenosis is another established factor that affects flow dynamics and alters WSS. It is thus not surprising that elevated PC-1 staining intensity was observed in atherosclerotic plaques of patients with arterial stenosis up to 50% at both arteries as well as with arterial stenosis up to 90% in one of the two carotid arteries. This observation confirms our previous findings that implicate PC-1 and PC-2 in the early endothelial fluid shear stress responses in healthy animals with partial carotid ligation^26^.

Interestingly, PC-1 expression was significantly increased in plaques from patients with diabetes mellitus, dislipidemia and hypertension. A possible role of PC-1in sterol regulation is supported by previous studies showing that loss of PC-1 or PC-2 results in dysregulated apolipoprotein expression in murine tissues via alterations in nuclear hormone receptors^45^. Moreover, an LDL receptor-like domain has been reported in PC-1 N- terminal with still unknown functions^46^.

Mutations in *PKD2* gene have been suggested to contribute to vascular hypertension, possibly due to dysregulation in the mechanism converting increased mechanical blood flow to cellular nitric oxide biosynthesis^19^. In addition, patients with ADPKD display major cardiovascular manifestations such as arterial hypertension^4^. Nevertheless, it is still unclear whether these manifestations are directly linked to aberrations of intracellular pathways involving PC-1 and PC-2, particularly in atherosclerotic plaques.

In our study a potential functional link of PCs presence in atherosclerotic plaques and intracellular signalling is suggested by the concomitant activation of the critical regulator of cell fate, p53. Polycystin signalling has been shown to activate p53, which in turn controls *PKD1* gene expression in an autoregulatory manner^47^. Furthermore, p53 has been found hyperexpressed in atherosclerotic lesions where it co-localises with increased Bax and p21 expression, providing a link between cell growth and apoptosis^31^. Biomechanical strain has been reported to induce activation of p53 in SMCs, the main cell component of vascular wall and subsequently to regulate their apoptosis^30^. In this study, activation of p53 was evident in stable and unstable atherosclerotic plaques following PC-1 upregulation. Activation of p38, a potential intermediate molecule of PC-1–p53 signalling was also elevated in atherosclerotic plaques indicating a possible link^30^. Previous studies suggest that p38 MAPK may be involved in transducing signals leading to cell death after mechanical stress by phosphorylating p53 in VSMCs^30^. p38 MAPK can phosphorylate both the N-terminal transactivation domain of human p53, as well as the C-terminus leading to conformational changes that expose the DNA-binding domain of the transcription factor^48^. The N-terminal phosphorylation of p53 leads to its release from the inhibitor MDM2. However, several p53 species exist that make possible other signals to concomitantly contribute to p53 activation and need further investigation.

In summary, the present study demonstrates that PCs are involved in the pathogenesis of atherosclerosis acting primarily as mechanosensor molecules detecting shear stress changes, but also as critical participants in atherosclerotic lesions implicated in inflammatory processes and plaque formation and evolution. A functional link with p53 activation as a mediator of PC intracellular signalling is proposed, providing a molecular mechanism of the increased vasoreactivity that underlies active coronary atherosclerotic plaque.

## Methods

### Cell culture of human endothelial cells

Human umbilical vein endothelial cells (HUVECs) were processed as previously described^49^. Cells were cultured in M199 medium (Gibco, Life Technologies) supplemented with 18% fetal bovine serum (FBS) (Gibco, Life Technologies), 1% penicillin-streptomycin (10,000 U/mL penicillin-10,000 μg/mL streptomycin), 2 mmol/L L-glutamine, 90 mg/ml heparin and low serum growth supplement. Cell cultures were maintained at 37 °C in a humidified atmosphere containing 5% CO_2_-95% air and grown to confluence.

### Shear stress application and measurements

For fluid shear stress experiments, HUVEC cultures in 25-cm^2^ flasks were placed in an incubating reciprocating platform shaker (Heidolph Promax 1020, Heidolph Instruments GmbH & Co, Germany) positioned inside the incubator. The numerical computations of the flow field and the distribution of the shear stress on the bottom wall of the cell culture flask and the cell monolayer, were studied with the commercial program Ansys-Fluent (Ansys Fluent Inc., Pennsylvania, USA). More specifically, the shear stress distribution on the cell monolayer was estimated from the equation:


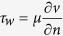


where μ is the constant fluid viscosity (37 °C water: 692 × 10^−6^ kg/ms) and ϑυ/ϑn is the normal velocity gradient at the wall. The maximum wall shear stresses during the cycle of reciprocating shakers were predicted using both numerical calculations and analytical flow theories.

Untreated cells or cells pretreated for 3 hours with an extracellular PC-1 inhibitory antibody (rabbit anti-IgPKD; 1:50, Genzyme Co., Boston, MA, USA) were equilibrated either in low oscillatory wall shear stress of 4 dynes/cm^2^ (corresponding to 60 rpm) or high oscillatory shear stress of 10 dynes/cm^2^ (corresponding to 110 rpm) in serum-free medium for 30 and 60 min, respectively. Control cells were identical passage HUVECs that were exposed to static conditions (0 dyne/cm^2^).

### Experimental animal model of low shear stress (LSS) induction

The partial carotid ligation animal model suitable for studies of early shear stress signalling on vascular endothelium was developed in our lab as previously described^26^. Briefly, six white Zealand rabbits, four-month-old (3.02 ± 0.21 kg) were anesthetized prior performance of a vertical midline incision (6 cm) at the neck. The left common carotid artery (LCCA) was identified and dissected for 1 cm free from surrounding tissue in two different areas. The first was at the most proximal part to the aortic arch and the second was 3 cm distally. Around the second segment an electromagnetic flow meter probe (2PSB-TS420; Transonic Systems) was placed and the mean flow signal was continuously displayed and recorded. At the first segment of the carotid artery a 4/0 suture (mersilk/ethicon) was passed around as a surgical loop. The LCCA was partially occluded with the aid of a small solid rubber rod which was placed between the external surface of the artery and the loop under constant monitoring of the volume flow. The incision was closed with a 3/0 Vicryl for the fascia and the subcutaneous tissue and 2/0 prolene for the skin. The volume flow at both right common carotid artery (RCCA) and LCCA and the partial occlusion of LCCA resulting in a LSS region upstream of the ligation, were also confirmed by ultrasonography. Upon recovery, the rabbits returned to its vivarium housing and given access to food and water. Five days later the animals were fasted for 12 hours prior to the ultrasound study and operation. An 8-cm vertical midline incision of the neck was performed; the two CCAs were identified, dissected and obtained. Euthanasia was performed by an I.V. injection of pentobarbital sodium solution (Dolethal) at a minimum dose of 120 mg/kg.

All animal experiments were performed in compliance with the guidelines and regulations set by the Ethics Committee of the University of Athens Medical School and according to the Guide for the Care and Use of Laboratory Animals (US NIH).

Wall shear stress (WSS) was estimated by using mean blood flow velocity (ū) and diastolic internal diameter (D_d_). Also maximal - peak WSS was estimated by the same formula using the maximal velocity (u_max_) and the systolic vessel diameter.

### Tissue specimens

Carotid plaques were prospectively collected from 69 random patients, who had internal carotid artery stenosis and underwent carotid endarterectomy. Demographic and clinicopathological data, medication, risk factors and vascular comorbidities were recorded (Table 3). Neurological evaluation of all patients was performed preoperatively in order to be assigned in the symptomatic or asymptomatic group. Symptomatic patients were classified based on the presence of stroke, transient ischemic attacks and amaurosis fugax. They also underwent a cerebral CT scan for identification of brain infarcts. Arteriography of the carotid bifurcation was performed in all patients for this study. The degree of stenosis was determined according to NASCET criteria^50^. Based on these measurements, stenotic lesions were divided into two subgroups (<90%, ≥90–99%).

All carotid plaque specimens were removed in the operating room and were divided into two portions. One portion was fixed immediately in 10% neutral-buffered solution with 4% formaldehyde for 24 hours, and embedded in paraffin for immunohistochemistry. The second portion was immediately stored at −80 °C for further analysis.

All experiments were carried out in accordance with the guidelines and regulations set by the Ethics Committee of the University of Athens Medical School. Furthermore, the entire study protocol was approved by the Ethics Committee of the University of Athens Medical School. Informed consent was obtained from all patients enrolled to the study.

### Reverse transcription-polymerase chain reaction (RT-PCR)

Total RNA was extracted using the TRIzol reagent according to the manufacturer’s protocol (Life Technologies-Invitrogen). Subsequently, cDNA was generated using the MMLV Superscript II RT according to the manufacturer’s instructions. *α-Tubulin* was used as a reference gene. PCR was performed in the linear range of amplification that would permit a quantitative assessment of expression levels. Primers specific for the human *PKD1* gene^51^ were as follows: forward 5′-CGC CGC TTC ACT AGC TTC GAC-3′; reverse 5′-ACG CTC CAG AGG GAG TCC AC-3′, giving a 260-bp product, for the human *PKD2* gene^19^: forward 5′-GCG AGG TCT CTG GGG AAC-3′; reverse 5′- TAC ACA TGG AGC TCA TCA TGC-3′, giving a 145-bp product and for the *α-tubulin* gene^19^: forward 5′-GCC AAC CAG ATG GTG AAA TG-3′; reverse 5′-GGT ACT CTT GGT CTT GAT GG-3′, giving a 125-bp product. The PCR conditions for *PKD1* were: 95 °C × 30 s, 55 °C × 30 s, 72 °C × 30 s, 35 cycles, for *PKD2*: 95 °C × 30 s, 52 °C × 30 s, 72 °C × 30 s, 35 cycles and for *α-tubulin* were 95 °C × 30 s, 50 °C × 30 s and 72 °C × 30 s. Amplified PCR products were electrophoresed on a 1.5% agarose gel containing ethidium bromide (0.5 μg/mL). Extracts from human thyroid artery (ThA) and human common carotid artery (HCCA) specimens were used as positive and negative controls, respectively. The experiments were repeated three times and the reported results are representative. Net band intensity (background-subtracted intensity) was normalized to values for *α-tubulin* (Quantity One Basic Software, Biorad Laboratories).

### Western immunobloting analysis

Upon completion of shear stress experiments, cells were washed twice with ice-cold PBS/50 mM NaF. The cell monolayer was scraped and centrifuged for 5 min at 1500 × *g*, 4 °C. Whole and nuclear protein extracts were separated, appropriately.

Whole cell protein extracts and atherosclerotic tissue specimens were homogenized in extraction buffer containing 10 mM Tris (pH 6.8), 5 mmol/L EDTA, 1% SDS, 5% glycerol, 50 mmol/L β-glycerophosphate, 1 mmol/L Na_3_VO_4_, 2.5 mM Na-pyrophosphate, 2 mmol/L leupeptin, 1 mmol/L aprotinin, 1 mmol/L DTT and 0.5 mmol/L PMSF. The homogenates were sonicated and centrifuged for 10 min at 12,000 rpm at 4 °C. Sample protein concentration was determined by the Bradford assay. Total protein lysates were resolved in 10% (25 μg) and 8% (50 μg) SDS-polyacrylamide gels and transferred to nitrocellulose membranes (Amersham Biosciences, Buckinghamshire, UK). Ponceau S staining of the membranes verified proper transfer of proteins. Membranes were incubated overnight at 4 °C with the primary antibodies anti-PC-1 (1:50, 7E12, sc130554, Santa Cruz Biotechnology, CA, USA), anti-PC-2 (1:50, PAB2306, Abnova, Europe), anti-phospho(p)-p53 (1:1000, phospho-Ser^15^, #9284, Cell Signalling Technology, Inc., MA, USA), anti-p53 (1:100, sc-47698, D07, Santa Cruz Biotechnology), anti-MDM2 (1:100, ab38619, Abcam, Cambridge, UK) and anti-phospho(p)-p38 (1:1000, phospho-p38 (Thr180/Tyr182) MAPK, #9215, Cell Signalling Technology). β-actin antibody (1:5000, MAB1501, Merck-Millipore, Darmstadt, Germany) was used as control to ensure equal sample loading. Band visualisation was performed using the enhanced chemiluminescence detection system (ECL, Amersham Biosciences, PA, USA) and quantification was performed based on β-actin bands using the Quantity One Basic Software (Biorad Laboratories). Protein extracts from ThA and HCCA specimens were used as positive and negative controls, respectively. The experiments were repeated at least three times and representative data are shown.

### Histological examination of tissue specimens

Haematoxylin and eosin staining was performed for histological evaluation of the specimens. Two pathologists, blinded to the clinical data, examined each specimen to assess atheromatous plaque morphology, using the American Heart Association classification of atherosclerotic plaques^52^. According to this classification, carotid plaques were assigned as fibroatherotic (type V) and complicated (type VI). The latter type included plaques with intraplaque hemorrhage, ulcer or thrombus, which were considered unstable.

### Immunohistochemical evaluation of tissue sections

The sections were deparaffinized and treated with the Dako Antigen Retrieval (pH 9) and Dako REAL™ EnVision™ Detection System (Dako Denmark A/S) according to the manufacturer’s instructions. Primary antibodies for anti-PC-1 (1:100, sc25570, Santa Cruz Biotechnology) and anti-PC-2 (1:50, PAB2306, Abnova) were used. For colour development, *3,3*-diaminobenzidine tetrahydrochloride (DAB) and hematoxylin as counterstain were used. Sections from human kidney and coronary artery were used as internal positive and negative controls, respectively to ensure optimal staining. Evaluation and quantification of staining (expression and intensity) was performed by an experienced pathologist (GA) using light microscopy.

Staining intensity was graded as 0 (negative), 1 (weak), 2 (moderate), and 3 (strong); percentage of positive cells examined was scored as 0 (negative), 1 (<20%), 2 (20–50%), 3 (>50%). The two scores were multiplied and the immunoreactive score (IRS) was determined: 0 as negative, values 1 as weak, values 2 as positive, and multiplication values 3, 4 as strongly positive.

### Statistical analysis

For the experimental animal model, continuous variables (i.e. hemodynamic and echo-doppler parameters) were compared between LCCA and RCCA using the non-parametric Wilcoxon matched pairs signed rank sum test.

The clinical samples were stratified as predefined groups of stable (Va, Vb) and unstable/complicated (VI) atherosclerotic plaques. Data are presented as mean ± standard error of mean (SEM). The significance of effects was determined by ANOVA or student’s *t* test using Statview 5.0 (Abacus Concepts, Cary, U.S.A.) software. *P* values less than or equal to 0.05 were considered statistically significant.

## Additional Information

**How to cite this article**: Varela, A. *et al.* Elevated expression of mechanosensory polycystins in human carotid atherosclerotic plaques: association with p53 activation and disease severity. *Sci. Rep.* **5**, 13461; doi: 10.1038/srep13461 (2015).

## Figures and Tables

**Table 1:** Immunohistochemical analysis of human atherosclerotic plaques.

	Va	Vb	VI
	n = 16	n = 38	n = 15
PC-1 expression	1.53 ± 0.20	1.82 ± 0.10	1.80 ± 0.91^**^
PC-2 expression	1.07 ± 0.10^†^	1.60 ± 0.10	1.20 ± 0.70
PC-1 staining intensity	1.73 ± 0.20^**^	1.76 ± 0.12^**^	1.60 ± 0.13
PC-2 staining intensity	0.93 ± 0.20	1.28 ± 0.10	1.33 ± 0.18
PC-1 IRS score (expression × intensity)	2.93 ± 0.65^*^	3.50 ± 2.29^*^	3.20 ± 0.55^*^
PC-2 IRS score (expression × intensity)	1.20 ± 0.24^†^	2.39 ± 0.29	1.86 ± 0.33

Mean ± SE

*p < 0.05, **p < 0.01 vs PC-2

^†^*p* < 0.01 vs PC-2 Vb

**Table 2:** PC-1 and PC-2 staining intensity values in atherosclerotic plaques from patients with specific risk factors.

	PC-1	PC-2
Diabetes	1.83 ± 0.15*	1.41 ± 0.13
Hypertension	1.67 ± 0.10***	1.36 ± 0.09
Dislipidemia	1.78 ± 0.10***	1.35 ± 0.09
Stenosis > 50% Left and Right Carotid Artery	1.72 ± 0.11**	1.33 ± 0.13
Stenosis > 90% Left or Right Carotid Artery	1.86 ± 0.12**	1.39 ± 0.14
Plaque histology status (stable or unstable)	1.75 ± 0.08**	1.42 ± 0.09

Mean values of intensity, mean ± SE

*p < 0.05, **p < 0.01, ***p < 0.005 vs PC-2

**Table 3:** Patients’ demographic and clinical data.

Patients	Overall	Symptomatic	Asymptomatic
Demographic Data	(n)	(n)	(n)
Male	58	31	27
Female	11	7	4
Mean age	68.7 ± 6.3		
Risk Factors			
Diabetes	20	14	6
Hypertension	49	24	25
Dislipidemia	42	34	8
Smoking	48	30	18
Ischemic Heart Disease	30	30	0
Clinical Symptoms			
Sroke	20	20	0
TIA	18	18	0
Amaurosis fugax	3	3	0
Angiographic carotid stenosis			
<90%	83	53	30
≥90%	33	33	0
CT Brain			
Positive	17	16	0
Negative	52	0	0
Plaque histopathology status			
Unstable	16	11	5
Stable	53	28	25
Medication			
β-blockers	22	8	14
Statins	26	8	18
CCBs	22	11	11
ACE inhibitors	44	26	18
